# 

**Published:** 1914-03-07

**Authors:** 


### CONTENTS.

PAGE

**Ambulance Problems Solved and Unsolved** ... 605

**Working Men and Hospital Legacies** ... ... 606

**Hospital and Institutional News** ... ... ... 607

A School for Secretaries—Rubber Floors for Edinburgh Royal Infirmary—The Marriage of Male Attendants—Mr. C. H. Wells and Guy's Hospital—The Bradford Infirmary Project—German and American Construction—Poor-Law Colony for Sane Epileptics—A Risk at the Start—A Mental Hospital Experiment—The Late Lord Strathcona and Forres Hospital—Sir Bland Sutton and Middlesex Hospital—Northampton Hospital and the "Letter" System—Dr. Chapple Attacks the "London."—A Scene in the House of Commons—The Lister Memorial—An Insolvent Insurance Committee—Lying-in Hospitals and the Insurance Act—Prisons as Sanatoria—A Model Society—A Hospital Threatened with Bankruptcy—Cyprus as a Health Resort—A Mental Deficiency Committee for London—Two Experimenters' Sudden Death—The Salaries of Poor-Law Clerks—Interesting Statistics from the London Hospital—Coming New Extensions at the "London"—This Week's Drug Market.

PAGE

**Modern Methods** ... ... ... ... ... 613

Recent Progress in Genito-Urinary Surgery.

**Reports on Hospitals of the United Kingdom**— 615

By SIR HENRY BURDETT, K.C.B., K.C.V.O

The Northampton General Hospital.

Letter from the Chairman.

[*See over.*]

### CONTENTS—(*continued*).

| | PAGE |
|---|---|
| **The Future of Mental Hospitals** ... ... ... | 614 |
| The Problem of Incipient Insanity. | |
| **The Soldiers' Daughters' Home, Hampstead** ... | 618 |
| A Grave Scandal: Where are the Health Authorities? | |
| **Hospitals from the Patients' Point of View** ... | 629 |
| Experiences in a Provincial Isolation Hospital. | |
| **The Future of St. Katharine's Hospital** ... | 620 |
| **Hospital Economics** ... ... ... ... ... ... | 621 |
| V. The Statistical Report of King Edward's Hospital Fund. | |
| **Round the World** ... ... ... ... ... ... | 622 |
| Fellow-Workers and their Doings. | |
| **The Institutional Library** ... ... ... ... | 623 |
| **The King and Queen at St. Thomas's Hospital** | 625 |
| Questions Raised at the Private Visit. | |
| **A Legacy Question at Cardiff** ... ... ... ... | 266 |
| **Beau Nash as a Hospital Officer** ... ... ... | 627 |
| Sidelights on the Mineral Water Hospital, Bath. | |
| **Institutional Needs** ... ... ... ... ... ... | 628 |
| **Public Pharmacists in Council** ... ... ... | 628 |
| **The Editor's Letter-Box** ... ... ... ... | 629 |
| "The Invention of Domiciliary Benefit." | |
| **The National Medical Union (Official)** ... ... | 630 |
| **The Institutional Worker** ... ... (Suppl.) | 1 |
| I.—Duties of the Male Servants. | |
| **Our Bureau of Information** ... ... ,, | 2 |
| Questions for March. | |

